# Glymphatic system dysfunction in recovered patients with mild COVID-19: A DTI-ALPS study

**DOI:** 10.1016/j.isci.2023.108647

**Published:** 2023-12-07

**Authors:** Lin Wu, Zhi Zhang, Xiao Liang, Yao Wang, Yuan Cao, Meng Li, Fuqing Zhou

**Affiliations:** 1 Department of Radiology, The First Affiliated Hospital, Jiangxi Medical College, Nanchang University, Nanchang, Jiangxi, China; 2 Jiangxi Province Medical Imaging Research Institute, Nanchang, Jiangxi, China; 3 Clinical Research Center For Medical Imaging, Nanchang, Jiangxi, China; 4 Department of Psychiatry and Psychotherapy, Jena University Hospital, Jena, Germany; 5 Center for Intervention and Research on adaptive and maladaptive brain Circuits underlying mental health (C-I-R-C), Jena-Magdeburg-Halle, Germany

**Keywords:** Clinical microbiology, Cognitive neuroscience, Microbiology, Neuroscience

## Abstract

Central nervous sequelae are often reported in recovered patients with COVID-19. It is not clear whether recovered COVID-19 patients have glymphatic impairment and clinical correlation. In this study, we demonstrated that mild COVID-19 patients experienced asymmetric bilateral glymphatic function decline after four months of recovery, and the decrease in glymphatic function was more obvious in older recovered patients. Our results further showed that recovered patients with right-sided glymphatic dysfunction experienced a greater proportion of cognitive decline (MoCA score <26) than patients with left-sided glymphatic dysfunction. With COVID-19 infection over 90% of the general population currently, future studies of cognitive disorders in the older population should consider the impact of COVID-19 infection.

## Highlights

• Bilateral glymphatic function decreased asymmetrically in patients with COVID-19
• Glymphatic dysfunction is more severe in elderly patients
• Decreased right DTI-ALPS index is more likely to show cognitive abnormalities

## Graphical abstract

**Figure:**
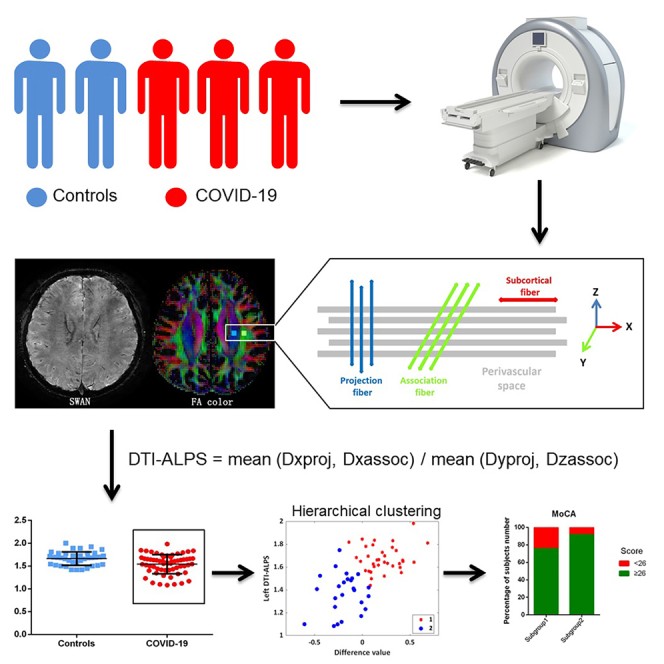

## Introduction

More than 90% of patients with COVID-19 survive, but varied types and frequencies of sequelae present after recovery, even in patients with mild symptoms after COVID-19 infection.^1^^,^^2^^,^^3^ The postsequelae symptoms range from neurocognition to respiratory and musculoskeletal symptoms, with predominant symptoms including headache, fatigue, breathlessness, arthralgia, sleep difficulties, and chest pain.^4^^,^^5^ Within one year of follow-up, neurological symptoms and psychological abnormalities are the most reported complications of sequelae.^4^^,^^6^^,^^7^

Conventional MRI findings often fail to explain these nonspecific symptoms. Few functional MRI studies have tried to explain the symptoms of depression and fatigue in COVID-19 patients.^8^^,^^9^ Over time, participants infected with COVID-19 showed persistent brain atrophy and a greater cognitive decline on average at the 3-year follow-up.^10^ COVID-19 can be neuroinvasive via hematogenous respiratory pathways and can also cause direct coronavirus neuroinvasion through olfactory receptors.^11^^,^^12^ The resulting changes in the limbic system, such as reduced gray matter thickness and tissue contrast in the orbitofrontal cortex and parahippocampal gyrus, may be the hallmarks of a degenerative spread of the disease through olfactory pathways of neuroinflammatory events. Moreover, researchers compared patients with other types of pneumonia and showed that these degenerative changes in the brain were specific to COVID-19 rather than a common feature of pneumonia.^10^

Additional studies postulate that this neurological syndrome may result from damage in olfactory sensory neurons, causing reduced outflow of cerebrospinal fluid through the cribriform plate and further leading to congestion of the glymphatic system with subsequent toxic build-up within the central nervous system.^13^ However, the relationship between COVID-19 infection and glymphatic function has not been clarified. If confirmed, the glymphatic system could serve as a potential target in combating sequelae of COVID-19, such as cognitive disorders.

The glymphatic system has been assessed *in vivo* in previous studies using dynamic contrast-enhanced MRI,^14^ intrathecal administration of gadolinium,^15^ and dynamic 11C-Pittsburgh Compound B positron emission tomography imaging techniques.^16^ However, the assessment of glymphatic function *in vivo* is still limited by the invasiveness of the current evaluation method. Recently, diffusion MRI has been proposed as a noninvasive method to quantify glymphatic function by calculating the diffusion tensor image metrics along the perivascular space (DTI-ALPS) index.^17^ The DTI-ALPS index was proven to be closely related to the classical detection methods of glymphatic clearance function.^15^ This method has been applied in studies on Alzheimer’s disease,^17^ Parkinson’s disease,^18^ ischemic stroke,^19^ sleep,^20^^,^^21^^,^^22^ idiopathic normal pressure hydrocephalus,^23^ tumor-associated cerebral edema,^24^ etc.

In this study, we hypothesized that outflow in the glymphatic system is impaired in recovered COVID-19 patients. To test this hypothesis, DTI-ALPS was employed to assess glymphatic system function and was further compared between healthy controls and recovered COVID-19 patients. Cluster analysis was performed to identify behavioral ratings or nonspecific symptoms associated with the DTI-ALPS index.

## Results

### Demographic and clinical features

Because most of the recruited patients had COVID infection with mild symptoms and no signs of pneumonia on computed tomography imaging, three patients were excluded from the final analysis because they were classified as having moderate or severe symptoms. Finally, a total of 61 patients with mild COVID-19 were included in this study.

The average age was 58.3 ± 10.3 years old, and 32 (52.5%) were females. Of these, eleven (18.0%) patients had a Montreal Cognitive Assessment (MoCA) score <26, indicating cognitive impairment. Nineteen (31.1%) had a Fatigue Severity Scale score >36, indicating fatigue. Seven (11.5%) had a Patient Health Questionnaire-9 score >10, indicating depression. Thirteen (21.3%) had a Pittsburgh Sleep Quality Index (PSQI) >8, indicating insomnia. Four (6.5%) had a Generalized Anxiety Disorder-7 score (GAD-7) >10, indicating anxiety. Four (6.5%) had a Hamilton Depression Scale score >17, indicating depression. Eight (13.1%) had a Hamilton Anxiety Scale score ≥14, indicating anxiety. Demographic and clinical score data from the study subjects are summarized in Table 1.

**Table 1:** Demographic, clinical examination, and clinical characteristics of recovered patients from mild COVID-19 and healthy controls

Characteristics	Recovered COVID-19 patients (N = 61)	Healthy controls (N = 38)	p value
Demographic data			
Age, years	43.7 ± 13.5	42.9 ± 7.9	0.738
Male, N (%)	29 (47.5)	17(44.7)	0.786
Education, years	11.9 ± 5.9	NA	NA
Clinical symptoms			
Fever, N(%)	53 (86.9)	NA	NA
Headache, N(%)	22 (36.1)	NA	NA
Cough, N(%)	34 (56.7)	NA	NA
Clinical data		NA	NA
Infection time to scan, days	80.8 ± 31.3	NA	NA
Respiratory symptoms disappear to scan, days	71.5 ± 33.7	NA	NA
Course of disease, days	9.3 ± 7.6	NA	NA
Clinical examination			
Montreal Cognitive Assessment score	27.3 ± 2.3	NA	NA
Fatigue Severity Scale score	26.3 ± 16.6	NA	NA
Pittsburgh Sleep Quality Index	4.4 ± 3.9	NA	NA
Patient Health Questionnaire-9 score	4.7 ± 4.8	NA	NA
Generalized Anxiety Disorder-7 score	2.8 ± 4.1	NA	NA
Hamilton Depression Scale	6.0 ± 6.5	NA	NA
Hamilton Anxiety Scale	4.9 ± 5.4	NA	NA

COVID-19, coronavirus disease 2019; NA, not applicable or not available.

### DTI-ALPS index comparison

To estimate the reproducibility of the DTI-ALPS index, we randomly selected 20 participants from the list of all enrolled participants. Regions of interest (ROIs) for each participant were repeatedly placed by the same radiologist (Z.Z.). 3 days later, we obtained the ALPS value and calculated the intraclass correlation coefficient. The intraclass correlation coefficient of the ROI definition was 0.88 (p < 0.001) in this study.

**Figure 1:**
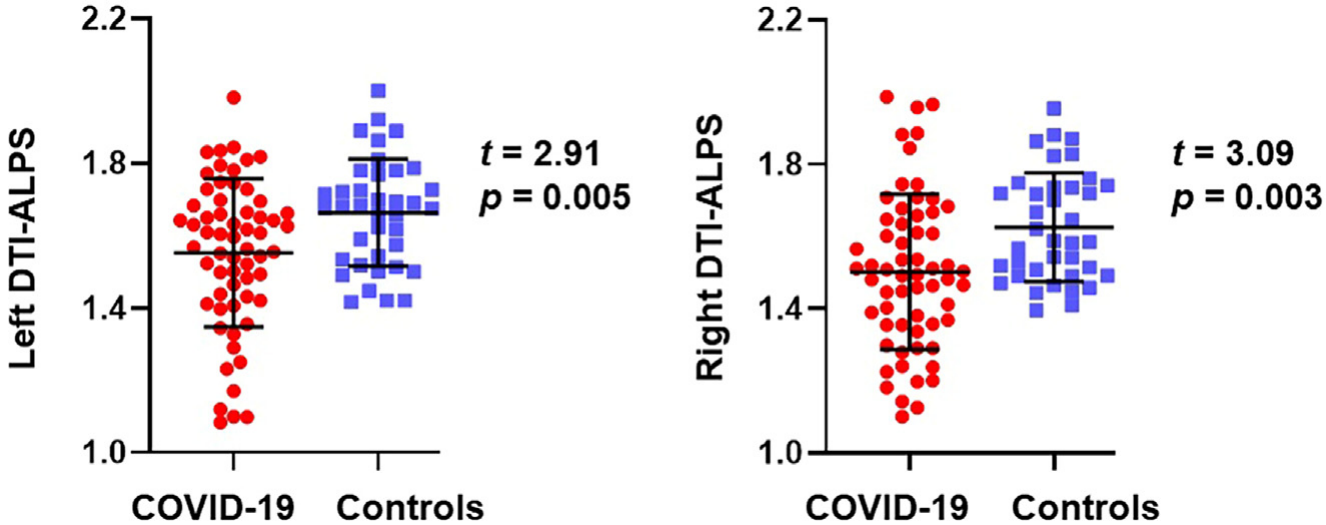
Differences in DTI-ALPS index between recovered patients with COVID-19 and healthy controls

Figure 1 presents the differences in the left and right DTI-ALPS index between recovered patients with COVID-19 and healthy controls. The DTI-ALPS index of recovered COVID-19 patients was significantly lower than that of healthy controls (left, 1.54 vs. 1.66, p = 0.005, Cohen’s d = 0.62; right, 1.51 vs. 1.62, p = 0.003, Cohen’s d = 0.66).

**Figure 2:**
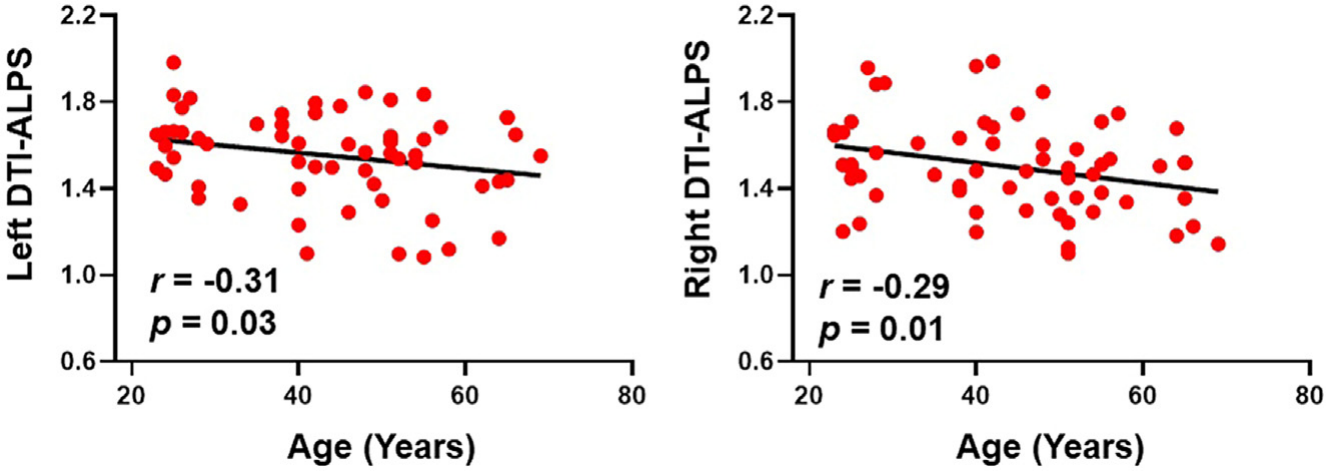
DTI-ALPS index and age-related scatter diagram in COVID-19 patients

**Figure 3:**
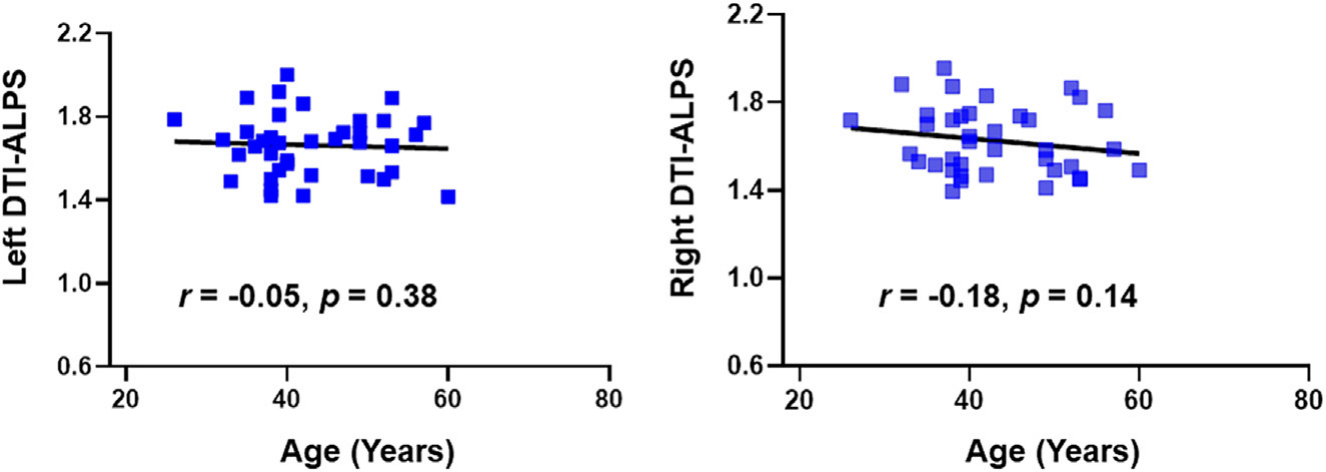
DTI-ALPS index and age-related scatter diagram in healthy controls

In the recovered COVID-19 patients, both left and right DTI-ALPS negatively correlated with age (Figure 2). There was no correlation for the DTI-ALPS index with education, course of disease, or clinical score (Table S1). In healthy controls, the bilateral DTI-ALPS index had no statistical correlation with age (Figure 3).

### Hierarchical cluster analysis in recovered COVID-19 patients

**Figure 4:**
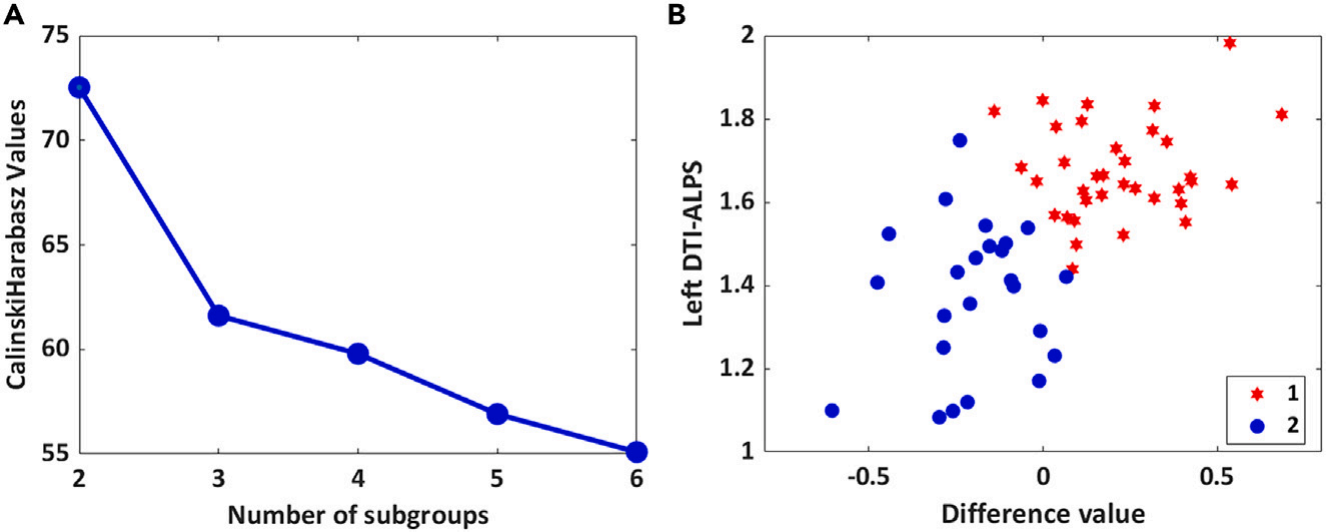
A data-driven cluster analysis (A) Evaluate the optimal number of subgroups using the Calinski-Harabasz cluster evaluation criterion. (B) The data contain left DTI-ALPS index and the difference between the left and right DTI-ALPS index.

**Figure 5:**
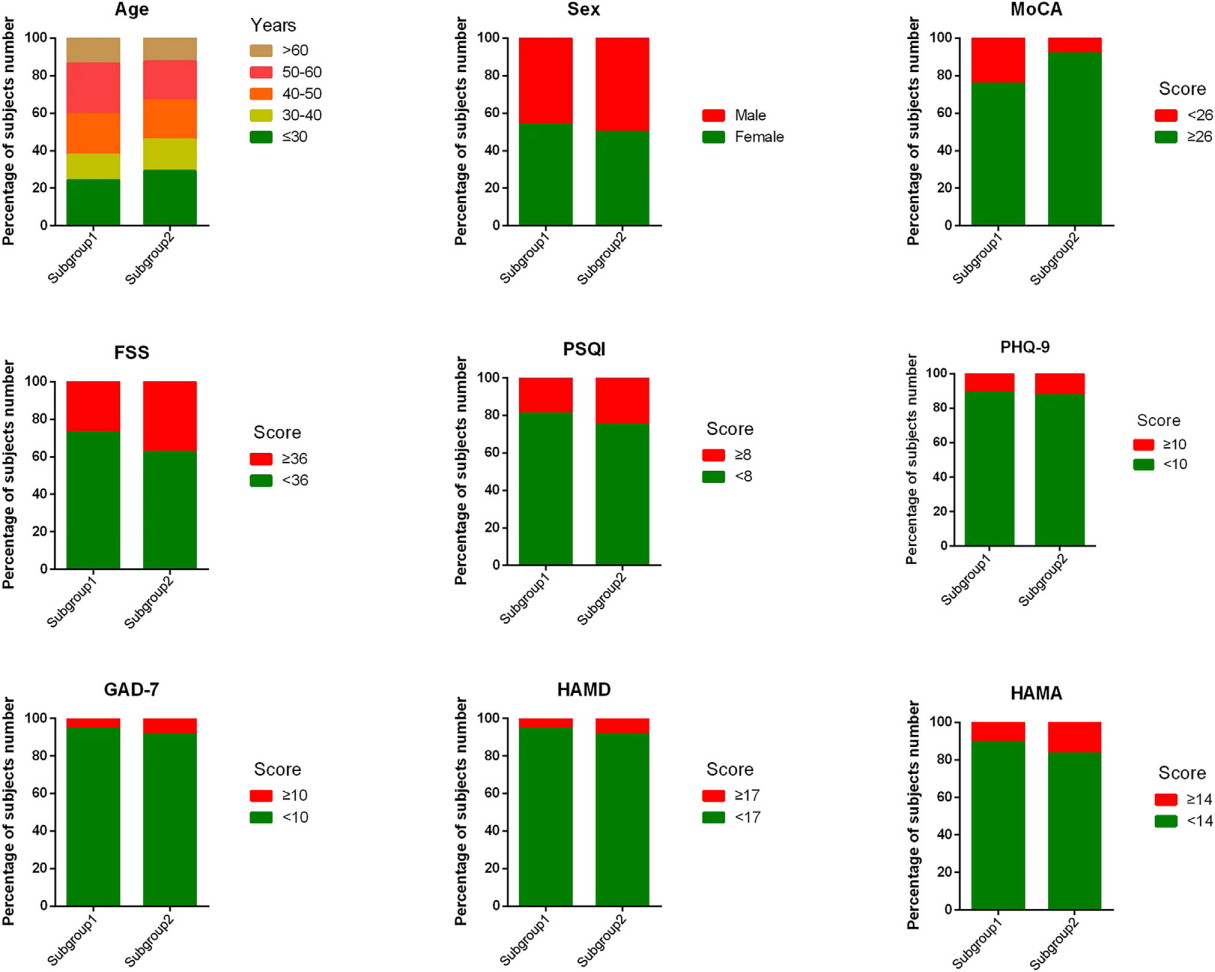
Proportion of demographic and clinical scores of subgroup 1 and subgroup 2 Abbreviations: DTI-ALPS, diffusion tensor image analysis along the perivascular space; MoCA, Montreal Cognitive Assessment; FSS, Fatigue Severity Scale; PSQI, Pittsburgh Sleep Quality Index; PHQ-9, Patient Health Questionnaire-9; GAD-7, Generalized Anxiety Disorder-7; HAMD, Hamilton Depression Scale; HAMA, Hamilton Anxiety Rating Scale.

To identify the behavioral ratings associated with the DTI-ALPS index, hierarchical cluster analysis was performed using two measured values (the left DTI-ALPS index and the difference between the left and right DTI-ALPS index). In addition to the previously described two indices, the left DTI-ALPS index and the left and right DTI-ALPS index were explored separately. Both resulted in an unconverging number of subgroups (Figure S1). On the basis of the Caliński-Harabasz pseudo-*F* index, the optimum number of clusters was determined to be two (Figure 4). The subgrouping dendrogram is shown in Figure S2. Here, the patients in this study could be further grouped into two subgroups (subgroup 1, n = 37, 60.7%; subgroup 2, n = 24, 39.3%). The DTI-ALPS index and demographic and clinical scores of subgroup 1 and subgroup 2 are described in Table 2 and Figure 5. Subgroup 1 showed a decline in the DTI-ALPS index on the right side, and a higher proportion of MoCA scores were lower than 26 (indicating cognitive abnormalities). Subgroup 2 showed a decline in the left ALPS index, a slightly longer course of the disease, and a higher proportion of fatigue, insomnia, depression, and anxiety. No significant difference was found between all the measured scores.

**Table 2:** The DTI-ALPS index, age, sex, and clinical scores of subgroup 1 and subgroup 2

Characteristics	Subgroup 1 (n = 37)	Subgroup 2 (n = 24)	p value
Left DTI-ALPS	1.66 ± 0.13	1.39 ± 0.20	**< 0.001**
Right DTI-ALPS	1.44 ± 0.18	1.60 ± 0.23	**0.005**
Age	44.5 ± 13.8	42.5 ± 13.3	0.566
Male, N (%)	17 (50.0%)	12(50.0%)	0.757
Education, years	11.8 ± 6.1	12.1 ± 5.8	0.815
Infection time to scan, days	80.0 ± 31.6	82.0 ± 31.3	0.809
Respiratory symptoms disappear to scanning time, days	71.2 ± 33.5	71.9 ± 34.6	0.929
Course of disease, days	8.8 ± 7.2	10.1 ± 8.3	0.552
Montreal Cognitive Assessment score	27.1 ± 2.4	27.7 ± 2.1	0.373
Fatigue Severity Scale score	25.8 ± 16.3	27.1 ± 17.2	0.765
Pittsburgh Sleep Quality Index	4.6 ± 4.8	4.8 ± 4.9	0.878
Patient Health Questionnaire-9 score	3.9 ± 3.2	5.3 ± 4.8	0.231
Generalized Anxiety Disorder-7 score	2.6 ± 3.8	2.9 ± 4.7	0.808
Hamilton Depression Scale	5.9 ± 6.6	6.1 ± 6.5	0.917
Hamilton Anxiety Scale	5.1 ± 5.1	4.5 ± 6.0	0.638

DTI-ALPS, diffusion tensor image analysis along the perivascular space.

We performed multiple linear regression analyses for both subgroups. It was found that the left DTI-ALPS index had effects on PSQI (reflecting sleep quality) and GAD-7 (reflecting anxiety status) in subgroup 1 (Table 3). No significant difference was found in subgroup 2 (Table S2).

**Table 3:** Multiple linear regression analysis in subgroup 1

Covariate	MoCA		FSS		PSQI		PHQ-9		GAD-7		HAMD		HAMA	
	β	*p*	β	*p*	β	*p*	β	*p*	β	*p*	β	*p*	β	*p*
Left DTI-ALPS	0.062	0.740	0.295	0.111	0.365	0.046	0.324	0.080	0.364	0.045	0.251	0.179	0.164	0.379
Right DTI-ALPS	−0.195	0.300	−0.233	0.204	−0.267	0.139	−0.164	0.367	−0.295	0.102	−0.135	0.465	−0.234	0.212

## Discussion

To the best of our knowledge, this is the first study to investigate the link between the glymphatic system and COVID-19 infection *in vivo*. In this study, our main findings include the following: (1) The left and right DTI-ALPS indices in recovered COVID-19 patients were lower than those in healthy controls at four months postinfection, regardless of sequelae symptoms. (2) Older people infected with COVID-19 had a greater decline in glymphatic function. (3) After hierarchical subgroup analysis, we found that the DTI-ALPS index on the left and right hemispheres decreased asynchronously in subgroup 1 and subgroup 2. Subgroup 1, with a lower right DTI-ALPS index, had a higher proportion of cognitive abnormalities. Multiple linear regression showed that the left DTI-ALPS had an effect on sleep quality and anxiety severity in subgroup 1.

COVID-19 may damage the brain via direct viral infection, systemic inflammation, and ischemia-hypoxia.^25^ These pathological changes also affect the structure and function of the glymphatic system. The brain-wide glymphatic pathway contains olfactory and perivascular space, which could remove waste products via the cerebrospinal fluid circulation. It has been proposed that cerebrospinal fluid clears metabolic waste from the interstitium through the perivascular space,^26^ particularly during rapid eye movement sleep. The partial fluid in the interstitium disperses toward the perineuronal spaces and then exits along the perineural sheaths of cranial nerves.^27^ When the COVID-19 virus invades the brain, damaged neurons or perineuronal spaces could reduce drainage efflux of interstitial fluid and further cause a disturbance between influx and efflux of fluid in the glymphatic pathway. It has been supported by a recent study that reported that the thickness of gray matter in the brain regions associated with smell, and memory decreased in COVID-19 patients, with a loss rate of 0.2%–2%. Due to impaired glymphatic clearance, the excessive perivascular accumulation of cell debris and metabolic wastes, in turn, aggravating neuroinflammation and neuronal death,^28^^,^^29^ might also accelerate the progression of brain inflammatory neurodegeneration.^30^ Ultimately, cognitive impairment may be the main long-term consequence of glymphatic failure.

In this study, we found that the ALPS index on both the left and right hemispheres in recovered patients was significantly lower than that in participants who had never been infected with COVID-19. A lower diffusion along the perivascular space indicates decreased glymphatic activity and impaired ability to clear metabolic wastes.^17^^,^^31^ As the first study to evaluate glymphatic system function in patients with COVID-19, our result is consistent with the atrophy of brain structure^10^^,^^32^ and particularly the disturbance of cerebrospinal fluid circulation.^13^ These potential brain changes may provide objective MRI evidence for delayed neurological sequelae. However, we must admit that the COVID-19 patient did not undergo any tests to directly assess the function of the glymphatic system prior to infection. For example, high-resolution MRI is used to evaluate glymphatic vessel structure and intrathecal gadolinium angiography is used to evaluate glymphatic function. When recruiting participants, we tried to exclude patients who may have had abnormal glymphatic function before infection with COVID-19. Additionally, our study found that a decreased DTI-ALPS index was associated with age in COVID-19 patients. Moreover, Douaudt et al. reported that the older the infected person is, the more severe the disease.^10^ We speculate that older people infected with COVID-19 are more susceptible to impaired glymphatic system function.

A data-driven approach based on the DTI-ALPS index data successfully identified two distinct groupings with our cohort. Subgroup 1 showed a decrease in the right ALPS index, while subgroup 2 showed a decrease in the left ALPS index. Regarding the lateralization of DTI-ALPS, the findings are controversial in the literature. Part of the reason lies in the fact that the ALPS index was measured only in the dominant hemisphere in some previous studies.^17^^,^^33^^,^^34^^,^^35^ It is reasonable to assume that the thicker fiber bundles in the left hemisphere were developed to minimize the possibility of losing perpendicularity between the fiber axis and the space around the vessels in a right-handed subject.^17^ The research found that the DTI-ALPS index of the left and right was similar or slightly higher on the left than on the right in healthy controls.^19^ Our results for healthy controls were consistent with previous observations, namely, the DTI-ALPS index was slightly higher in the left hemisphere (1.66 ± 0.15) than in the right hemisphere (1.63 ± 0.15). Nonsymmetric decline in the left and right DTI-ALPS index may indicate brain injuries in different stages in patients with COVID-19. Subgroup 1, with a decrease in the right DTI-ALPS index, had a larger proportion of cognitive decline, although there was no significant difference in proportion between the two subgroups in our study. The lack of significance of subgroup differences might relate to the included subjects based on community recruitment, and most patients had mild symptoms, including fever, dry cough, fatigue, myalgia, loss of smell and taste, and a slight decline in memory. Additionally, we must keep in mind that severe and critically ill patients were not included because of the limited number of participants.

Considering the correlation between the DTI-ALPS index and age, we avoided the multicollinearity of independent variables. Only the left and right ALPS indices were used as independent variables for multiple linear regression analysis. In subgroup 1, we found that the left DTI-ALPS index had a significant effect on sleep quality. Several studies have shown that sleep quality is significantly related to glymphatic function.^20^^,^^22^^,^^36^ Sleep fragmentation and altered sleep architecture can potentially hinder the glymphatic system. However, the causal relationship between sleep disorders and glymphatic decline needs further study. According to the clustering results, the decline in glymphatic function among subgroups was lateralized. It is warranted to investigate the indicative functional differences between the left and right DTI-ALPS indices in future studies. Although we found that left DTI-ALPS had a trend level of significant effect on the GAD-7 in subgroup 1, given the nature of the severity of infection in all patients and that only 6.6% of the patients had typical anxiety symptoms (GAD ≥10), there is a lack of evidence to conclude that the left glymphatic anxiety-related outcome is a characteristic of patients with COVID-19.

The glymphatic system could facilitate waste clearance from the brain parenchyma by the rapid exchange between cerebrospinal fluid and interstitial fluid. The characterization of this exchange process *in vivo* could be observed upon serial MRI following intrathecal gadolinium-based contrast agent injection.^37^ Intrathecal administration of gadolinium is an invasive operation requiring multiple MRI scans with fixed time intervals, which is difficult to apply widely in clinical practice. With a one-time scan and without any contrast agent, noninvasive DTI-ALPS can be applied in larger populations in the clinic. The findings further support its accuracy by showing that the ALPS index is significantly related to the indicators of intrathecal injection of gadolinium to detect glymphatic clearance function *in vivo*.^15^ The second advantage of using DTI-ALPS is that measuring water diffusion in the direction of the perivascular space has high repeatability and reliability and is not affected by the shape of the ROI (such as square, cube, or sphere).^38^ Nevertheless, the robustness of manual delineation on the DTI-ALPS ROIs was further confirmed by the high intrarater correlation analysis in this study (r = 0.88).

In conclusion, we found that, with or without sequelae, the DTI-ALPS index is lower in recovered COVID-19 patients within four months, which might reflect impairment of glymphatic function. The decline in glymphatic function is more pronounced when the infected person is older. Hierarchical subgroup analysis showed that the decline in the DTI-ALPS index was nonsynchronized in the two hemispheres. Patients with a decreased right DTI-ALPS index showed a higher proportion of cognitive abnormalities. Future studies on cognitive impairment in older adults should rule out the effect of COVID-19 infection.

### Limitations of the study

This study has a few limitations to bear in mind when generalizing our findings. First, although the ALPS index was highly correlated with glymphatic system function as measured by intrathecal injection of gadolinium-based contrast agents, the DTI-ALPS index only represents instantaneous glymphatic function at the time of MRI scanning in the awake state.^15^ Glymphatic efficacy during sleep is superior to that during wakefulness.^39^ Second, our study found that the ALPS index of recovered patients with COVID-19 was significantly reduced compared with that of uninfected patients within four months. The symptoms of sequelae after recovery of COVID-19 patients are varied, and patients often have more than two symptoms.^2^^,^^3^^,^^5^^,^^40^ This may be another factor in why the unilateral glymphatic decline was not statistically associated with clinical scores. Therefore, it is necessary to enlarge the sample size further to identify the clinical significance of the lateralized decline in glymphatic function. Third, no educational data were collected from healthy controls in our study. Despite these limitations, this study can characterize glymphatic changes at four months after recovery in a population of mild COVID-19 patients.

## STAR★Methods

### Key resources table

**Table:** 

REAGENT or RESOURCE	SOURCE	IDENTIFIER
**Software and algorithms**		

MATLAB 2020B	MathWorks	https://www.mathworks.com/products/MATLAB.html
DSI studio	DSI Studio	http://dsi-studio.labsolver.org
MRICroGL	NITRC	https://www.mccauslandcentre.sc.edu/mricrogl
SPSS	IBM Software	https://www.ibm.com/products/spss-statistics
GraphPad Prism	PraphPad Software	https://www.graphpad.com
DTI-ALPS index	Toshiaki Taoka	N/A

### Resource availability

#### Lead contact

Further information and requests for resources and reagents should be directed to and will be fulfilled by the lead contact, Fuqing Zhou (ndyfy02301@ncu.edu.cn).

#### Materials availability

This study did not generate new unique reagents.

#### Data and code availability

Any additional information required to reanalyze the data reported in this paper is available from the lead contact upon request. This article does not produce any original code related to research or data analysis.

### Experimental model and subject details

#### Participants

We recruited 61 subjects who recovered from COVID-19 by the following inclusion criteria: (1) confirmed SARS-CoV-2 infection within the past four months and recovered; (2) ages 20–70; (3) right-handed; (4) no other diagnosed primary or successive neurological diseases; 5) no history of insomnia prior to infection; 6) no prior history of mental illness, such as depression, anxiety, mania, etc. and 7) no contraindications to MRI (i.e., claustrophobia, pregnancy or breastfeeding). The exclusion criteria were as follows: (1) incomplete imaging examination and related neuropsychological quantum examination; and (2) MRI showing apparent abnormal brain structure or signal. We recruited 29 male and 32 female COVID-19 patients. We also included 38 age- and sex-matched healthy individuals from August 2021 to October 2022 as healthy controls with no history of medical or neurological disorders. After the COVID-19 pandemic lockdown was abolished in December 2022, the infection rate was more than 80% in the general population, and it was difficult to recruit healthy controls without COVID-19 infection afterwards. All subjects were Han Chinese from Nanchang, Jiangxi, China.

#### Ethics statement

The Institutional Review Board of the First Affiliated Hospital of Nanchang University approved this study (ethical number 2023-018). All subjects gave written informed consent prior to study participation.

#### Clinical assessment

At the time of study enrolment, all recovered COVID-19 patients underwent a clinical examination, as follows: 1) Montreal Cognitive Assessment (MoCA) score, with a total score of 0–30 points. Generally, a score greater than or equal to 26 points is normal, while a score less than 26 points indicates cognitive impairment. 2) Fatigue Severity Scale (FSS): the minimum score is 9, the maximum score is 63, and a score greater than or equal to 36 indicates fatigue. 3) Patient health questionnaire-9 (PHQ-9), a total score of 0–27 points, with a score greater than or equal to 10 indicating depression. 4) Pittsburgh Sleep Quality Index (PSQI), a total score of 0–21 points, with a score greater than or equal to 8 indicating insomnia. 5) Generalized anxiety disorder-7 (GAD-7), a total score range is 0–21 points, with a score greater than or equal to 10 indicating anxiety. 6) The Hamilton Depression Scale (HAMD) has a total score of 0–52 points, with a score greater than or equal to 17 indicating depression. Moreover, 7) the Hamilton Anxiety Scale (HAMA) has a total score of 0–56 points, with a score greater than or equal to 14 indicating anxiety.

According to the "Diagnosis and Treatment Protocol for Novel Coronavirus Infection (Trial 10th edition)" published on the website of China’s National Health Commission, the severity of COVID-19 infection is usually divided into four levels, namely mild, moderate, severe, and critical, according to clinical and imaging manifestations.

### Method details

#### MRI acquisition

All of the MRI scans were performed using the same scanner (3.0T, Signa Prioneer, GE Healthcare, USA, 24-channel head coil). During scanning, each subject remained in the supine position, put on earplugs, and used foam pads to reduce head movements. DTI was conducted using spin-echo single-shot echo-planar pulse sequences with a total of 32 different diffusion directions: repetition time (TR)/echo time (TE) = 11015/73.5 ms, slice thickness = 2.5 mm, acquisition matrix = 128 × 128, field of view = 224 × 224 mm^2^, and *b*-value = 1000 s/mm^2^. T2 star weighted angiography (SWAN) with TR/TE = 43.2/4.0 ms, slice thickness = 2 mm, acquisition matrix = 220 × 220 mm^2^, and flip angle = 20^o^ was also used.

#### DTI-ALPS index calculation

The data were processed using DSI studio software (version 2021). The process is as follows: 1) read the digital imaging and communications in medicine (DICOM) file and perform quality inspection; 2) set up a mask to filter out the background region, increase the reconstruction efficacy, and facilitate further visualization; 3) perform reconstruction with the DTI method to characterize the major diffusion direction of the fibres, and 4) diffusion images for each subject were subsequently registered to SWAN to accurately select the brain areas with veins and perivascular spaces running over the x-axis. Over the slice, using the colour-coded principal diffusion direction map, we drew two 3-voxel (approximately 5.4 mm) square regions of interest (ROIs): one ROI was drawn over the area of projection fibre with fibre axes corresponding to the z-axis and the other over the area of associative fibre with fibre axes corresponding to the y-axis. The DTI-ALPS index is calculated as follows: DTI-ALPS = mean(Dxproj, Dxassoc)/mean (Dyproj, Dzassoc). A low DTI-ALPS index indicated impaired glymphatic function according to the definition.^17^

#### Subgroup analyses

The core of subgroup analysis is to select appropriate indicators or distance metrics to group objects based on the differences and similarities of data. Using the SPM toolbox in MATLAB, we calculated the Caliński-Harabasz pseudo-F index for groupings between 2 to 6 subgroups to identify the optimum number of subgroups. Visual inspection of the subgrouping dendrogram confirmed the number of people in each subgroup. After hierarchical subgrouping, the proportions of demographic and clinical dysfunction among the different subgroups are presented in Figure S2 and Table 2.

### Quantification and statistical analysis

Comparisons were conducted using the chi-square test for categorical variables and independent samples *t* test or the Mann‒Whitney test for continuous variables according to a normal distribution tested with the Shapiro‒Wilk test. Continuous variables are represented as the mean values with standard deviations. Statistical significance was defined when the p value was less than 0.05. One-tailed Pearson correlation was used for correlation analysis. All statistical analyses were performed using SPSS software version 22 (IBM, Chicago, IL).
