# Supplementary material for: Glymphatic system dysfunction in recovered patients with mild COVID-19: A DTI-ALPS study
Source: iScience. 2023 Dec 7;27(1):108647. doi: 10.1016/j.isci.2023.108647 (PMC10753064; doi:10.1016/j.isci.2023.108647)
Supplement: Document S1. Figures S1 and S2 and Tables S1 and S2 [file mmc1.pdf]

iScience, Volume 27

Supplemental information

Glymphatic system dysfunction in recovered patients with mild COVID-19: A DTI-ALPS study

Lin Wu, Zhi Zhang, Xiao Liang, Yao Wang, Yuan Cao, Meng Li, and Fuqing Zhou

Table S1 Correlation between demographic, clinical data and DTI-ALPS index, related to Figure 2

**Characteristics**	**Left DTI-ALPS**		**Right DTI-ALPS**	
	r	***p value***	***r***	***p value***
Education (years)	0.211	0.103	0.204	0.115
Course of disease (days)	-0.057	0.663	-0.063	0.630
Respiratory symptoms disappear to scan (days)	-0.237	0.066	-0.159	0.220
Montreal cognitive assessment score	0.021	0.873	0.098	0.451
Fatigue severity scale score	0.125	0.338	0.001	0.994
Patient health questionaire-9 score	0.109	0.405	0.019	0.886
Pittsburgh sleep quality index	-0.097	0.457	-0.108	0.405
Generalized anxiety disorde-7 score	0.120	0.358	-0.034	0.794
Hamilton depression scale	0.132	0.310	0.001	0.992
Hamilton anxiety scale	0.105	0.419	-0.141	0.278

Figure S1 Evaluate the optimal number of subgroups, related to Figure 4

Evaluate the optimal number of subgroups using the Calinski-Harabasz subgrouping evaluation criterion. To identify the behavioural ratings associated with the DTI-ALPS index, hierarchical cluster analysis was performed using two measured values (the left DTI-ALPS index, and the difference between the left and right DTI-ALPS index). Both resulted in an unconverging number of subgroups.

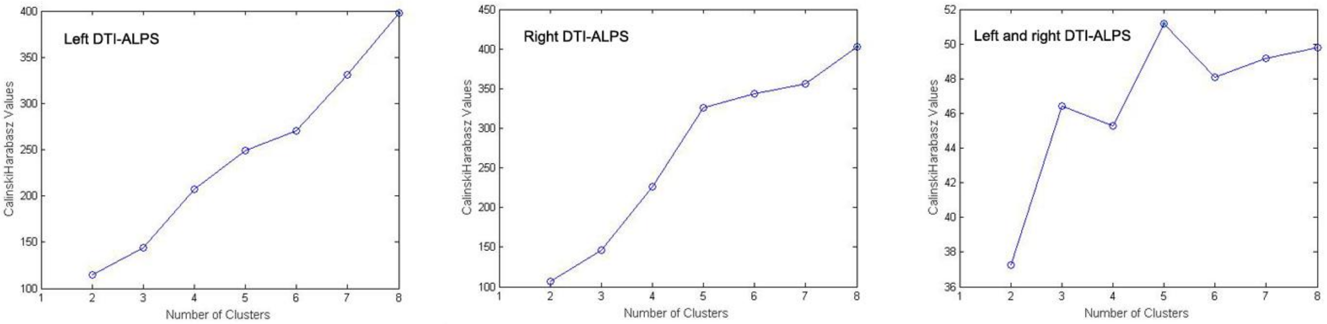

Figure S2 Dendrogram of hierarchical cluster analysis, related to Figure 4

The subgrouping dendrogram is shown. Here, the patients could be further grouped into two subgroups (subgroup 1, n = 37, 60.7%; subgroup 2, n = 24, 39.3%).

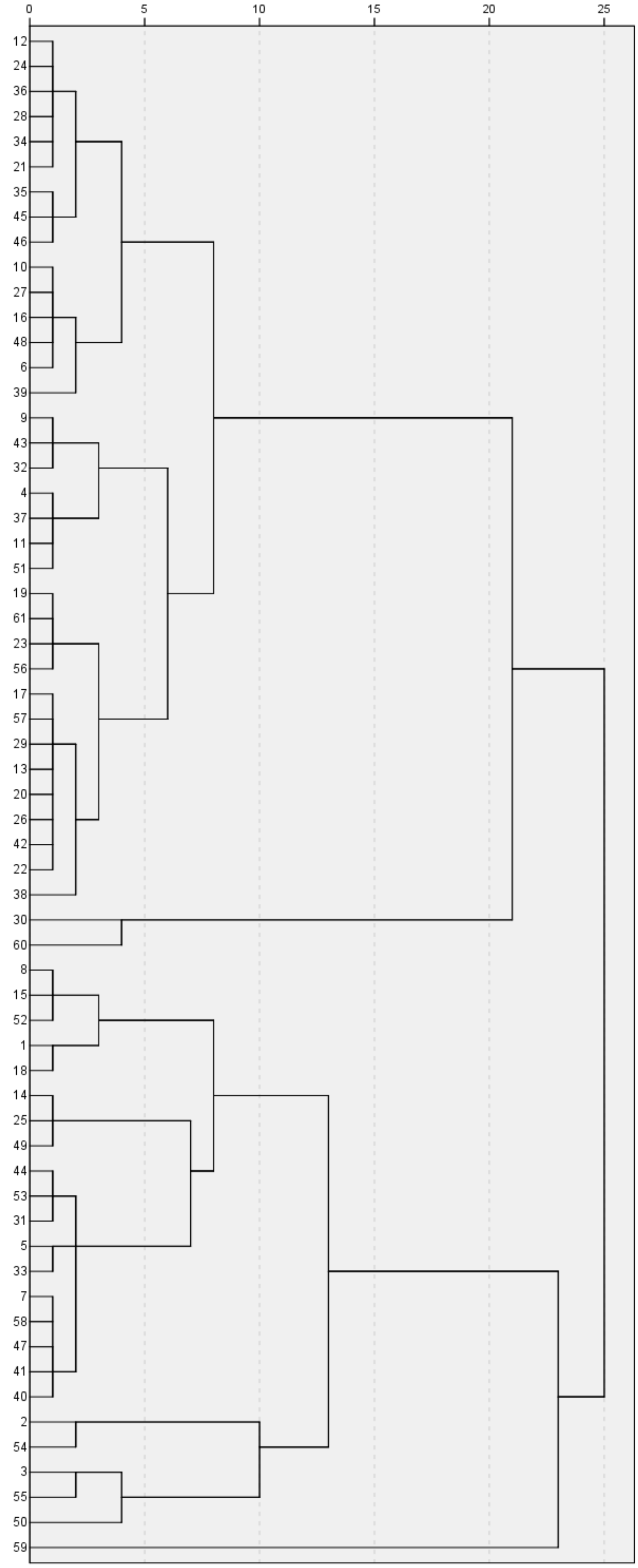

Table S2 Multiple linear regression analysis in Subgroup 2, related to Table 3

**Covariate**	**MoCA**		**FSS**		**PSQI**		**PHQ-9**		**GAD-7**		**HAMD**		**HAMA**	
	**β**	***P***	**β**	***P***	**β**	***P***	**β**	***P***	**β**	***P***	**β**	***P***	**β**	***P***
Left DTI-ALPS	0.008	0.980	0.285	0.395	0.093	0.777	0.041	0.904	0.230	0.495	0.368	0.272	0.403	0.226
Right DTI-ALPS	0.381	0.232	-0.117	0.726	-0.310	0.351	0.033	0.922	-0.127	0.705	-0.249	0.454	-0.394	0.236

Abbreviations: DTI-ALPS, diffusion tensor image analysis along the perivascular space; MoCA, montreal cognitive assessment; FSS, fatigue severity scale; PSQI, pittsburgh sleep quality index; PHQ-9, patient health questionaire-9; GAD-7, generalized anxiety disorde-7; HAMD, hamilton depression scale ; HMMA, hamilton anxiety scale.
